# Functional Differences between Periportal and Perivenous Kupffer Cells Isolated by Digitonin-Collagenase Perfusion

**DOI:** 10.1186/1476-5926-2-S1-S34

**Published:** 2004-01-14

**Authors:** Igor Bykov, Petri Ylipaasto, Leena Eerola, Kai O Lindros

**Affiliations:** 1 Depatment of Alcohol Research, National Public Health Institute, POB 33, 00251 Helsinki, Finland; 2 Department of Virology, National Public Health Institute, POB 33, 00251 Helsinki, Finland

## Introduction

Liver injury commonly appears in a zonated fashion within the liver acinus. Thus many hepatotoxins primarily damage the perivenous (centrilobular, PV) region [1]. It is now well established that Kupffer cells (KCs) play a crucial role as initiators and mediators of the inflammatory process in the liver through the release of inflammatory and immunomodulatory mediators. However, the role of KCs in initiating regional damage is not established. Several studies based on immunohistochemistry have shown that the KCs in the periportal (PP) area are larger and cell size separation based on elutriation suggest that they are more active in phagocytosis [2-5]. The evidence suggesting that the smaller KCs in the centrilobular region would be more active in cytokine production is not clear-cut [3,4].

The aim of this study was to isolate KCs from different parts of the liver acinus and compare their capacity for phagocytosis and for secretion of key molecules mediating cytotoxicity.

## Methods

To isolate intact KCs originating from either the PP or the PV region, the digitonin-collagenase perfusion technique, previously used to study functional differences of hepatocytes, was applied [6]. Briefly, livers from male Wistar rats were perfused, and then region-specific cell destruction was achieved by infusion of a short pulse of 3.5 micromolar digitonin solution, that only partly penetrated into the sinusoids. For isolation of PP cells, digitonin was infused via the upper vena cava. For isolation of PV cells, PP digitonin infusion was used. Residual digitonin solution was immediately flushed out from the opposite direction. This was followed by conventional collagenase perfusion. The hepatocyte fraction was removed by centrifugation (2 – 30 sec – 50 g) and KCs were purified by Percoll gradient centrifugation. The cells were plated on plastic dishes, incubated at 37 degrees C in an atmosphere of 5% CO_2 _for 45 min. and non-adherent cells removed by washing. The viability of the cells, determined by Trypan blue exclusion or by fluorescence labeling, routinely exceeded 95%. PP and PV cells were always prepared the same day and identically treated during all steps.

For phagocytosis, cells were exposed to FITC-labelled zymosan particles for 10–30 min. The uptake was estimated by confocal microscopy (Leica, TCS NT) after nuclear labeling using 1 micromolar ethidium homodimer-1 (Molecular probes, E-1169) and quantitated by a fluorescence counter (Victor 2, Wallac, Finland).

The effect of LPS (100 ng/ml, 4 h) on cytokine, PGE_2 _and NO production was determined in freshly purified or in 24 h cultured KCs. TNF-alpha, IL-1 beta, IL-10 and PGE_2 _were determined using commercial ELISA kits and NO using the Griess reagent.

## Results

To confirm successful removal of unwanted cell populations, the activity of ALT, an established PP marker, was determined from corresponding hepatocyte fractions. The ALT activity in PP preparations was 1.7 times higher than in PV cell preparations (p &lt; 0.01), thus confirming the different acinar origin of the KC.

Phagocytosis of KCs was monitored by confocal microscopy. KCs from the PP region routinely had taken up more FITC labelled zymosan particles both after 10 and 30 min exposure as compared to PV cells. Fluorescence after 30 min exposure to FITC-zymosan was quantitated using a multilabel fluorescence counter. Phagocytosis was 2.2 times higher (p &lt; 0.05) by KCs from the PP region than that by PV preparations.

Basal and LPS-stimulated production of TNF-alpha, IL-10, IL-1 beta, PGE_2 _and NO during a 4-hour incubation was determined both from freshly isolated cells and after culture for 24 hours. Overnight culture was found to dramatically influence the response of both KC populations to TNF-alpha production. Freshly isolated KCs produced more than 10 times more TNF-alpha than after 24 hours of culture. Interestingly, freshly isolated KCs from the PP region secreted significantly more TNF-alpha (+73 %; p &lt; 0.05) than PV cells. This difference was also seen in the presence of LPS, which only modestly increased secretion in both preparations. In contrast, after overnight culture, LPS markedly stimulated TNF-alpha production by both cell preparations. The fold-stimulation was more marked in PV KCs (26-fold) as compared to PP cells (11-fold). IL-1 beta production was also significantly higher in PP than in PV KCs, but only in freshly isolated cells. In contrast to TNF-alpha, more IL-1 beta was produced by overnight cultured cells and LPS had no marked effect. Neither culture time nor addition of LPS had any significant effect on IL-10 production and there was only a tendency for a PP>PV difference in basal IL-10 production by fresh cells. The PP-PV pattern of NO and PGE_2 _production was opposite to that found for pro-inflammatory cytokines: freshly isolated PV KCs produced more NO and PGE_2 _than PP cells (p &lt; 0.05) while a tendency towards an opposite pattern was observed after overnight culture. LPS had no significant effect.

## Discussion

We found that KCs isolated from the PP region phagocytosed FITC-labelled zymosan particles more actively as compared to PV cells. This confirms previous indirectly obtained evidence [2-5], demonstrating that periportal KCs indeed have a crucial role in the first line defenses against foreign particles.

The production of cytokines and other mediators by the KC subfractions and their response to LPS was different in pre-and postculture conditions. Thus TNF-alpha production was more than 10 times higher in freshly isolated KCs as compared to overnight cultured cells. This probably reflects the stress of the recent isolation procedure and explains the modest extra effect of LPS addition. The low LPS response may also be related to reduced post-isolation function of CD14 receptors. Cultured cells recover and their low basal TNF-alpha production allows a marked LPS response.

Investigating the KC responses both during pre- and postculture conditions seems important. Thus our data on freshly isolated cells indicate that PP KCs are more active in producing pro-inflammatory cytokines (TNF-alpha, IL-1 beta). This is in accordance with an elutriation study, where the production of these cytokines was more active by larger KCs [7]. On the other hand, another study reported that smaller KCs (presumably representing PV cells) responded to LPS challenge by producing more IL-1 beta than larger cells [4]. However, this discrepancy may be due to different culture conditions. Indeed, in the present study, TNF-alpha measurements showed that overnight cultured PV KCs responded stronger to LPS (26-fold increase) than PP cells (11-fold). Interestingly, LPS at the dose used (100 ng/ml) did not significantly stimulate IL-1 beta or IL-10 production even after overnight culture.

We previously showed a higher CD14 receptor mRNA expression in the PV as compared to PP region [8]. It is feasible that the stronger LPS response of overnight cultured PV KCs as compared to PP cells could be due to recovered activity of CD14 receptors in these cells. This could support the concept of a more active role of the smaller PV KCs in the inflammatory process.

In contrast to cytokine secretion, freshly isolated PV KCs produced more NO and PGE_2 _as compared to PP cells. This finding agrees with an elutriation study showing that smaller KCs displayed the highest level of NO secretion [9]. Studies indicating that both NO and PGE_2 _affect TNF-alpha synthesis [10,11] suggest that in PV KCs the higher NO and PGE_2 _release could be associated with their lower TNF-alpha production.

In conclusion, Kupffer cell isolated from the periportal region are the most active macrophages in phagocytosis but the acinar heterogeneity of basal and LPS-stimulated secretory responses by Kupffer cell subfractions are different before and after culture. Before culture periportal Kupffer cells produce more pro-inflammatory cytokines while after culture the perivenous cells respond stronger to LPS.
